# A Bearing Fault Diagnosis Method Based on Dual-Stream Hybrid-Domain Adaptation

**DOI:** 10.3390/s25123686

**Published:** 2025-06-12

**Authors:** Xinze Jiao, Jianjie Zhang, Jianhui Cao

**Affiliations:** College of Mechanical Engineering, Xinjiang University, Urumqi 830017, China; 107552304265@stu.xju.edu.cn (X.J.); 107552204151@stu.xju.edu.cn (J.C.)

**Keywords:** domain adaptation, fault diagnosis, transfer learning, pseudo-labeling, dual-stream network, bearings

## Abstract

Bearing fault diagnosis under varying operating conditions faces challenges of domain shift and labeled data scarcity. This paper proposes a dual-stream hybrid-domain adaptation network (DS-HDA Net) that fuses CNN-extracted time-domain features with MLP-processed frequency-domain features for comprehensive fault representation. The method employs hierarchical domain adaptation: marginal distribution adaptation (MDA) for global alignment and conditional domain adaptation (CDA) for class-conditional alignment. A novel soft pseudo-label generation mechanism combining Gaussian mixture models (GMMs) with the Mahalanobis distance provides reliable supervisory signals for unlabeled target domain data. Extensive experiments on the Paderborn University and Jiangnan University datasets demonstrate that DS-HDA Net achieves average accuracy values of 99.43% and 99.56%, respectively, significantly outperforming state-of-the-art methods. The approach effectively addresses bearing fault diagnosis under complex operating conditions with minimal labeled data requirements.

## 1. Introduction

With the advent of the Industry 4.0 era, the deep integration of modern information technology and manufacturing has significantly propelled advancements in the machinery manufacturing and industrial production sectors. Rotating machinery, as a core asset in industrial production, is widely used in critical fields such as power, oil and gas, chemical engineering, and metallurgy. Its stable operation is directly related to production efficiency [1] and equipment safety. Among these components, bearings, as key parts of rotating machinery, undertake the crucial function of supporting rotating shafts, and their health status has a decisive impact on the safe operation of the entire system. However, the working environment of bearings is often complex and harsh. Statistics show that approximately 40% of faults in rotating machinery equipment are caused by bearing failures. Therefore, accurate and real-time detection and diagnosis of bearing faults can not only effectively prevent equipment failures but also significantly reduce maintenance costs and production downtime—both of which are crucial for ensuring the smooth progress of machinery manufacturing and industrial production. Challenges in adversarial domain adaptation for such tasks have also been noted.

**Figure 1:**
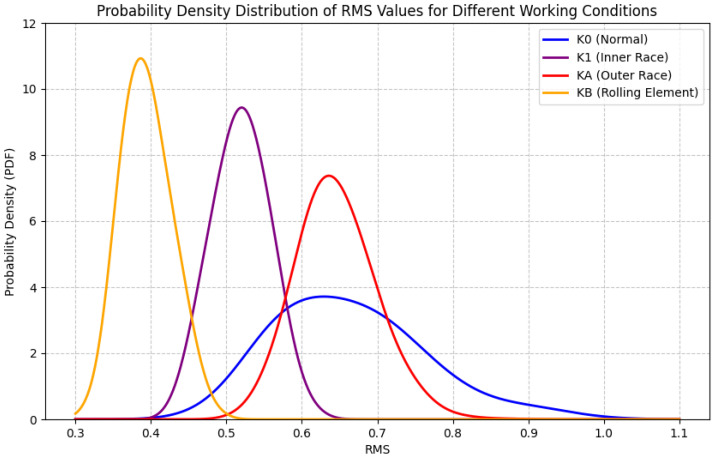
Probability density distribution of RMS values of vibration signals for different bearing states. (The figure shows that the distributions of various states overlap, indicating that a single traditional statistical feature may be insufficient for reliable fault classification, especially when working condition changes cause further distribution drift).

Traditional bearing fault diagnosis methods primarily rely on signal processing techniques and shallow machine learning algorithms, such as time-domain analysis, frequency-domain analysis, and time-frequency analysis. These methods can effectively extract fault features and perform classification under ideal conditions. However, in the complex working conditions of industrial sites, the operating environment of bearings is often affected by load changes, speed fluctuations, and noise interference, resulting in significant differences in fault data distribution and difficulties in feature extraction. The diagnostic performance of traditional methods is significantly limited, especially in scenarios with insufficient data or significant changes in working conditions, where their accuracy and robustness fail to meet practical needs. For example, even commonly used statistical features, such as the root mean square (RMS) value of signals, may exhibit significant overlap in probability density distributions for different fault types (as shown in Figure 1), which undoubtedly increases the difficulty of achieving precise diagnosis relying solely on simple features. Hybrid models combining CNN and MLP have been explored to address these feature extraction challenges. Meanwhile, in specific scenarios with scarce labeled data or limited sample sizes, some research has focused on condition-based diagnostic approaches. These methods often do not solely rely on large-scale data-driven techniques but attempt to combine prior knowledge [2], signal processing techniques, or shallow models to establish condition recognition rules or models for specific equipment or operating conditions. For instance, diagnosis might be performed by meticulously analyzing energy changes at specific fault frequencies, kurtosis indicators, or envelope spectrum features, often in conjunction with classifiers like expert systems or support vector machines (SVMs). However, while these condition-based methods may show some effectiveness on small datasets, their generalization ability is often limited to specific operating conditions and fault modes, making them difficult to adapt to complex [3], variable operating environments, and diverse fault manifestations, and they typically rely heavily on domain-specific expertise.

In recent years, with the rapid development of big data and artificial intelligence technologies, data-driven intelligent fault diagnosis methods have gradually become a research hotspot. Deep learning-based models, such as convolutional neural networks (CNNs) and recurrent neural networks (RNNs), have significantly improved fault diagnosis performance by automatically extracting deep features. However, these methods typically assume that the training data (source domain) and test data (target domain) have the same distribution. In actual industrial scenarios, due to changes in working conditions (such as variations in speed, load, or environmental conditions) [4], the data distributions of the source and target domains often exhibit significant differences. Acquiring sufficient labeled samples covering all working conditions is both time-consuming and expensive. This phenomenon is known as domain shift, which can cause a severe decline in the generalization ability of models trained on the source domain when applied to the target domain, thereby reducing the accuracy and reliability of fault diagnosis [5]. Spiking neural networks have also been investigated for bearing fault diagnosis. Indeed, early work using multi-layer neural networks highlighted the variability in fault characteristics under different conditions.

To address the problem of domain shift, domain adaptation (DA) techniques have emerged, aiming to transfer knowledge learned from a labeled source domain to an unlabeled or sparsely labeled target domain. DA methods are diverse and can be broadly categorized. Discrepancy-based methods form one major category, focusing on directly minimizing the distribution divergence between the source and target domains. A common example is Maximum Mean Discrepancy (MMD), which aligns marginal distributions by minimizing the difference of mean embeddings in a reproducing kernel Hilbert space (RKHS), though it may sometimes overlook class-specific structures. Other approaches in this category, such as correlation alignment (CORAL), aim to align second-order statistics like covariance matrices. Adversarial-based adaptation methods constitute another significant category, exemplified by domain adversarial training networks (DANNs). DANNs introduce a domain [6] discriminator that competes with the feature extractor in a minimax game to learn domain-invariant features. While effective, the training of basic DANN can be unstable and may sometimes lead to confusion about class information. To mitigate such issues, subsequent developments like conditional domain adversarial networks (CDANs) incorporate class predictions into the adversarial learning process to encourage more [7] discriminative domain-invariant features. Reconstruction-based methods, often employing autoencoders, are also utilized to learn a shared latent space where domain-specific characteristics are preserved while domain discrepancies are reduced, or to enforce domain invariance through reconstruction losses, thereby facilitating knowledge transfer. Although these DA techniques have achieved considerable progress, effectively preserving class discriminability during feature alignment, handling unstable training dynamics, and adapting to the diverse complexities of real-world applications remain persistent challenges in the field. Successful DA applications typically necessitate carefully designed adaptive strategies and an in-depth understanding of the specific problem domain.

Although existing methods have made some progress, they still face challenges: (1) A single type of feature (e.g., only time-domain or only frequency-domain) may be insufficient to fully capture fault information under complex working conditions. (2) Global alignment (like MMD) may disrupt class structures [8], while conditional alignment is sensitive to the quality of pseudo-labels. (3) Hard pseudo-labels cannot reflect prediction uncertainty and may introduce noise. Transfer learning from laboratory bearings to real-world applications like locomotive bearings highlights these practical difficulties. Furthermore, considering the correlation of multi-stage degradation is important for remaining useful life prediction, a related field.

To address these challenges [9], this paper proposes a novel dual-stream hybrid-domain adaptation network (DS-HDA Net) for bearing fault diagnosis. The core ideas of this method are as follows:

1. **Dual-stream feature fusion:** Concurrently use CNN to process raw time-domain vibration signals and MLP to process extracted frequency-domain features (such as frequency band energy, envelope spectrum peak values), then fuse the information from both to obtain more robust and comprehensive feature representations.
2. **Hybrid-domain adaptation:** Apply MDA on the fused features for global alignment to reduce overall domain differences; apply CDA on the model output logits for conditional alignment, utilizing soft pseudo-labels to preserve class discriminability.
3. **GMM-based soft pseudo-labeling:** Utilize GMMs trained on source domain fused features, combined with model predictions and the Mahalanobis distance, to generate more reliable soft pseudo-labels for target domain samples, providing effective unsupervised learning signals.

Experimental results on public datasets show that DS-HDA Net achieves significant performance improvements in cross-condition bearing fault diagnosis tasks compared to various benchmark methods. The transfer from scientific test rigs to industrial applications is a key goal.

## 2. Key Theories and Technologies

Rotating machinery, especially bearings, operates under diverse conditions due to speed, load, and environmental changes. Data from one condition (source domain, DS) and another (target domain, DT) often have distinct statistical distributions. This domain shift can degrade machine learning model performance when models trained on DS data are applied to DT data. Domain adaptation (DA) addresses this by transferring knowledge from labeled DS to unlabeled or sparsely labeled DT, improving model generalization across conditions. This paper focuses on unsupervised DA, where DT labels are unavailable during training. Bridging this domain gap is key to developing robust fault diagnosis systems.

### 2.1. Maximum Mean Discrepancy (MMD)

Maximum mean discrepancy (MMD) is a fundamental non-parametric metric for measuring the distance between two probability distributions, P and Q, based on samples drawn from them. MMD maps distributions to a reproducing kernel Hilbert space (RKHS) H using a kernel function ϕ(·). The squared MMD is defined as the squared distance between the mean embeddings of the two distributions in this RKHS [10]. Targeted transfer learning via a distribution barycenter medium is also an advanced concept.

(1) $$MMD^{2}\left(P,Q\right)=\left|\right|E_{x∼P}\left[\phi \left(x\right)\right]-E_{y∼Q}\left[\phi \left(y\right)\right]{\left|\right|}_{H}^{2}$$

In practice, given samples ${\left\{x_{i}\right\}}_{i=1}^{n_{S}}$ from P and samples ${\left\{y_{j}\right\}}_{j=1}^{n_{T}}$ from Q, an empirical estimate of the (biased) squared MMD can be calculated using a kernel function $k\left(\;\cdot \;,\;\cdot \;\right)={\langle \phi \left(\;\cdot \;\right),\phi \left(\;\cdot \;\right)\rangle }_{H}$:

(2) $$MMD^{2}\left(\left\{x_{i}\right\},\left\{y_{j}\right\}\right)=\frac{1}{n_{S}^{2}}\sum_{i=1}^{n_{S}}\sum_{i^{\prime }=1}^{n_{S}}k\left(x_{i},x_{i^{\prime }}\right)+\frac{1}{n_{T}^{2}}\sum_{j=1}^{n_{T}}\sum_{j^{\prime }=1}^{n_{T}}k\left(y_{j},y_{j^{\prime }}\right)-\frac{2}{n_{S}n_{T}}\sum_{i=1}^{n_{S}}\sum_{j=1}^{n_{T}}k\left(x_{i},y_{j}\right)$$

Commonly, Gaussian (RBF) kernels, or a mixture of RBF kernels with different bandwidths, are used. Minimizing MMD encourages the learned feature representations to [11] be statistically similar between the source and target domains, thereby reducing the domain shift.

### 2.2. Covariance Alignment

While MMD primarily aligns the first-order statistics (means) of distributions in RKHS, aligning second-order statistics, such as covariance, can provide complementary information about the data structure and further reduce domain discrepancy [12]. Covariance alignment aims to match the covariance matrices of feature representations in the source domain ($\Sigma_{S}$) and target domain ($\Sigma_{T}$). A common way to measure the difference between covariance matrices is to compute the squared Frobenius norm of their difference.

(3) $$\left|\right|\Sigma_{S}-\Sigma_{T}{\left|\right|}_{F}^{2}$$

Minimizing this difference helps to make the feature representations across domains have similar dispersion and orientation, contributing to more robust domain adaptation.

### 2.3. Hybrid-Domain Adaptation: MDA and CDA

This paper employs a hybrid adaptation strategy that combines global distribution alignment with class-conditional alignment. Such hierarchical strategies are part of ongoing research in intelligent fault diagnosis and aim for more fine-grained adaptation.

#### 2.3.1. Marginal Distribution Adaptation (MDA)—Global Alignment

To reduce the overall domain gap at the feature level, marginal distribution adaptation (MDA) [13] is applied. In this model, MDA aligns the global distributions of the fused features extracted from both source and target domains. This is achieved by minimizing a loss function that combines MMD (Equation (2)) and covariance alignment (Equation (3)). This alignment operates on the fused features obtained from the CNN and MLP streams (details of feature fusion are provided in Section 3.2.3, Section 3). The MDA loss function is defined as follows:

(4) $$L_{MDA}=\lambda_{mmd}\;\cdot \;MMD^{2}\left(f_{fused_{S}},f_{fused_{T}}\right)+\lambda_{cov}\;\cdot \;||\mathrm{Cov}\left(f_{fused_{S}}\right)-\mathrm{Cov}\left(f_{fused_{T}}\right){\left|\right|}_{F}^{2}$$

where $f_{fused_{S}}$ and $f_{fused_{T}}$ represent the fused features of the source and target domains, respectively. $\lambda_{mmd}$ and $\lambda_{cov}$ are weight hyperparameters. This loss encourages the fused representations across domains to be globally indistinguishable.

#### 2.3.2. Conditional Distribution Adaptation (CDA)—Class-Conditional Alignment

Although global alignment reduces the overall domain difference, it might inadvertently mix features from different classes if the conditional distributions P(Y|X) differ significantly between domains. Conditional distribution adaptation (CDA) addresses this issue by aligning feature distributions [14] conditioned on class labels [15]. This helps to preserve the discriminative structure learned in the source domain when transferring knowledge to the target domain. In this model, CDA operates on the logits (the output of the final classification layer before Softmax activation). It is achieved by minimizing a per-class MDA loss (again, combining MMD and covariance alignment applied to logits), using source domain true labels ($Y_{S}$) and target domain soft pseudo-labels (${\hat{Y}}_{T}$, as generated in Section 2.5).

(5) $$L_{CDA}=\sum_{c=1}^{C}\mathrm{MDA}(logits_{S}|Y_{S}=c,logits_{T}\left|{\hat{Y}}_{T}=c\right)$$

where $logits_{S}$ and $logits_{T}$ are the model’s logit outputs for the source and target domain samples, respectively, and C is the total number of classes. The MDA function operates on the logits corresponding to each class c.

### 2.4. Gaussian Mixture Model (GMM)

A Gaussian mixture model (GMM) is a probabilistic model that represents the data distribution as a weighted sum of multiple Gaussian components [16]. It is suitable for modeling complex data distributions where a single class might exhibit multiple modalities or clusters. The probability density function of a GMM is as follows:

(6) $$p\left(x\right)=\sum_{k=1}^{K}\pi_{k}N\left(x|\mu_{k},\Sigma_{k}\right)$$

where K is the number of components, $\pi_{k}$ denotes the mixture weights (satisfying $\sum_{k=1}^{K}\pi_{k}=1$, $\pi_{k}\geq 0$), and $N(x|\mu_{k},\Sigma_{k})$ denotes the probability density function of the k-th Gaussian component with mean $\mu_{k}$ and covariance $\Sigma_{k}$. GMM parameters $\left(\pi_{k},\mu_{k},\Sigma_{k}\right)$ are typically estimated using the expectation–maximization (EM) algorithm [17]. While GMMs are a standard modeling tool, their specific application in this work for refining pseudo-labels in conjunction with the Mahalanobis distance (as detailed next) is a key aspect of the proposed method.

### 2.5. Soft Pseudo-Label Generation Based on GMM and the Mahalanobis Distance Adjustment

In unsupervised domain adaptation (UDA), reliable pseudo-labels for unlabeled target samples are vital for effective conditional distribution alignment (CDA) and supervision, as traditional “hard” pseudo-labels can introduce noise from uncertain predictions. This paper proposes a soft pseudo-label generation strategy using Gaussian mixture models (GMMs) with the Mahalanobis [18] distance adjustment, aiming for more dependable supervisory signals that reflect prediction uncertainty. The process begins by training a multi-component GMM (GMMc) for each class c with labeled source domain fused features to model each class’s probability distribution. Then, for each target sample, its fused features are used to calculate its likelihood (p gmm,c) under every class GMM, establishing an initial data distribution-based prior. This approach leverages the principle of learning transferable features, which is foundational to deep adaptation networks.

To further improve the quality of these GMM-based priors, the Mahalanobis distance is introduced for probability adjustment. Specifically, for a target sample’s fused feature $f_{T}$, its minimum Mahalanobis distance to the centers ($\mu_{k}$) of all Gaussian components within each class GMM ($GMM_{c}$) is calculated using Equation (7):

(7) $$d_{M}\left(f_{T},\mu_{k},\Sigma_{k}\right)=\sqrt{{\left(f_{T}-\mu_{k}\right)}^{T}\Sigma_{k}^{-1}\left(f_{T}-\mu_{k}\right)}$$

This distance, $\min\_dist_{gmm,c}\left(f_{T}\right)=\min_{k\in GMM_{c}}d_{M}\left(f_{T},\mu_{k},\Sigma_{k}\right)$, reflects the proximity of the sample to the core of the class distribution as modeled by the GMM components [19]. An exponential decay function based on this minimum Mahalanobis distance is then used to adjust the previously calculated GMM likelihoods (Equation (8)):

(8) $$p_{adjusted,c}=p_{gmm,c}\;\cdot \;\exp\left(-\beta \;\cdot \;\min\_dist_{gmm,c}\left(f_{T}\right)\right)$$

where β is a sensitivity parameter. This adjustment effectively reduces the probability weights of samples that are far from the core region of any component of a class GMM. Domain-adaptive neural networks for object recognition also explore similar adaptation principles.

**Figure 2:**
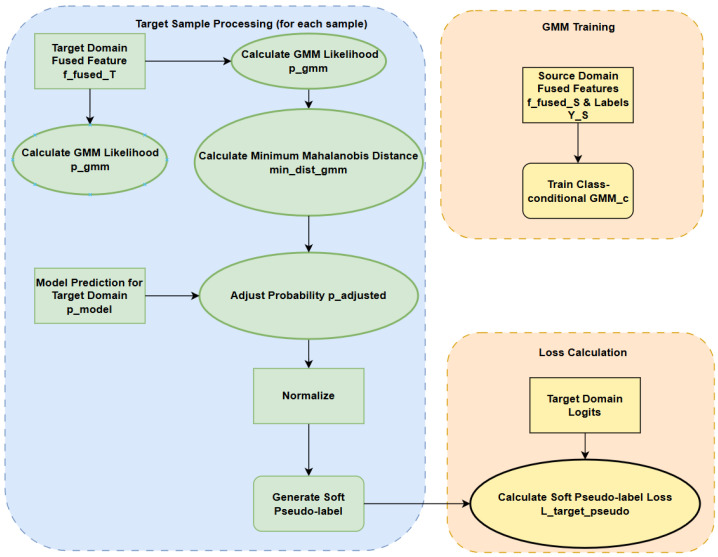
This figure presents a flowchart of the soft pseudo-label generation strategy, integrating Gaussian mixture models (GMMs) and the Mahalanobis distance adjustment. It delineates three primary stages: (i) ‘GMM training’ using source domain data; (ii) ‘target sample processing’, where target domain fused features are transformed into soft pseudo-labels via computations including the GMM likelihood, the minimum Mahalanobis distance, probability adjustment, fusion with model predictions, and normalization; and (iii) ‘Loss Calculation’, illustrating how target domain logits and the generated soft pseudo-labels are utilized to compute $L_{target\_pseudo}$.

Finally, this Mahalanobis distance-adjusted, feature distribution-based probability ($p_{adjusted,c}$) is weighted and fused with the [20] model’s own Softmax prediction probability ($p_{model,c}$) for the target sample (derived from $logits_{T}$), as shown in Equation (9):

(9) $$P_{final,c}=\alpha \;\cdot \;p_{model,c}+\left(1-\alpha \right)\;\cdot \;p_{adjusted,c}$$

where α is a weighting factor. The resulting $P_{final}$ vector, after normalization to sum to 1, serves as the final soft pseudo-label. This soft label [21], which integrates model prediction confidence with GMM-based distributional priors (corrected by the Mahalanobis distance), provides a more robust target distribution for calculating the class cross-entropy loss for the target domain samples (Equation (10)):

(10) $$L_{target\_pseudo}=-\frac{1}{N_{T}}\sum_{j=1}^{N_{T}}\sum_{c=1}^{C}P_{final,j,c}\;\cdot \;\log\left(\mathrm{softmax}{\left(logits_{T,j}\right)}_{c}\right)$$

thereby guiding the model to better adapt to the target domain data. The complete process of this GMM and the Mahalanobis distance-adjusted soft pseudo-label generation strategy is intuitively illustrated in Figure 2. Unsupervised domain adaptation is a core theme in deep transfer networks.

### 2.6. I-Softmax Loss Function

To enhance the discriminative ability of features learned from the source domain [22], thereby improving their transferability, this paper adopts the I-Softmax loss function instead of the standard Softmax cross-entropy loss for source domain classification. I-Softmax belongs to a family of margin-based Softmax loss functions designed to increase inter-class distance and reduce intra-class variance in the learned embedding space.

These loss functions typically modify the logits before the Softmax calculation. The specific implementation in the provided code (class ‘I_Softmax’) adjusts the logit value corresponding to the true class label ($Y_{S}$) for a given source sample. If $logits_{S,i}$ is the logit vector for the *i*-th source sample and $Y_{S,i}$ is its true label, the logit component $logits_{S,i,Y_{S,i}}$ corresponding to the true class is modified using scalar parameters *m* and *n*. This modification, denoted here as $logits_{S,i,Y_{S,i}}^{\prime }$, aims to create a decision margin. The other logit components ($logits_{S,i,j}$ where $j\neq Y_{S,i}$) remain unchanged. The I-Softmax loss is then computed as the standard cross-entropy loss applied to these modified logits:

(11) $$L_{source\_cls}=\mathrm{CrossEntropyLoss}\left(\mathrm{softmax}\left(logits_{S}^{\prime }\right),Y_{S};m,n\right)$$

By effectively enforcing a margin, I-Softmax encourages the model to learn more discriminative and compact features for each class in the source domain. This is beneficial for domain adaptation tasks, as more separable features are often more robust to the domain shift. Adversarial discriminative domain adaptation also focuses on learning discriminative features.

## 3. The Proposed Transfer Learning Model: DS-HDA Net

### 3.1. DS-HDA Net Framework

To address the domain shift challenge in bearing fault diagnosis caused by varying operating conditions, this study proposes a dual-stream hybrid-domain adaptation network (DS-HDA Net). The core innovation of this model lies in its dual-stream architecture design, aimed at fusing the advantages of different data representations; one stream employs a convolutional neural network (CNN) to directly process raw time-domain vibration signals for automatic learning of hierarchical features, while the other utilizes a multi-layer perceptron (MLP) to process manually designed frequency-domain features (such as frequency band energy ratios and envelope spectrum peak information) to capture explicit physical meaning. Through a feature fusion module, information from both streams is effectively integrated to obtain a more comprehensive and robust representation of bearing states, laying a solid foundation for subsequent domain adaptation and classification tasks. The use of transformers, like bottleneck transformers, is also an emerging trend in visual recognition that shares principles of feature extraction.

**Figure 3:**
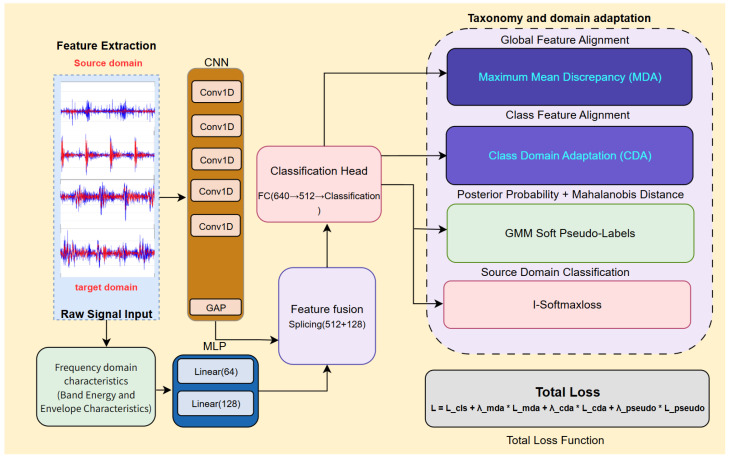
Overall architecture of the DS-HDA Net model. Showing feature extraction (CNN for time-domain, MLP for frequency-domain), feature fusion, and classification/adaptation modules (MDA, CDA, GMM soft pseudo-labeling, I-Softmax source classification, and the total loss function).

Building upon this dual-stream architecture, DS-HDA Net further integrates a hybrid-domain adaptation strategy and advanced supervised learning mechanisms. Specifically, the model applies marginal distribution adaptation (MDA) at the fused feature level for global domain alignment to reduce overall domain differences; simultaneously, it applies conditional domain adaptation (CDA) at the classifier output logit level for fine-grained class-conditional alignment to maintain discriminative structure during cross-domain transfer. To effectively guide target domain learning, a soft pseudo-label generation technique based on Gaussian mixture models (GMMs) and the Mahalanobis distance adjustment is adopted to provide reliable supervisory signals for unlabeled target samples. Additionally, source domain classification employs I-Softmax loss, aiming to enhance the intra-class compactness and inter-class separability of features. Finally, by end-to-end optimizing a combined objective function that includes source domain classification loss, target domain pseudo-label loss, and MDA/CDA domain adaptation losses, the network is driven to learn domain-invariant and highly discriminative feature representations. Time series transformers have also been applied to machinery fault diagnosis. Figure 3 illustrates the overall architecture of the DS-HDA Net.

### 3.2. Dual-Stream Feature Extractor

The feature extractor aims to capture information from different perspectives of the vibration signal. Auto-encoding variational Bayes is a related generative modeling approach [23].

#### 3.2.1. CNN Stream for Raw Signals

This stream uses a convolutional neural network to process raw one-dimensional time-series vibration data. As shown in Table 1 (see Table 1 for details), the CNN architecture comprises multiple convolutional modules. Each module typically includes Conv1d, BatchNorm1d, ReLU, and MaxPool1d layers. An AdaptiveAvgPool1d layer is used at the end of the CNN stream to aggregate features along the time dimension, generating a fixed-size vector representation. This stream directly learns hierarchical features from the raw signal waveform. Domain adversarial graph convolutional networks offer another perspective on feature learning under variable conditions.

#### 3.2.2. Frequency-Domain Feature Extraction and MLP Stream

As a complement to time-domain analysis, this stream processes manually designed frequency-domain features. According to the code implementation, the following 11 features are extracted for each signal segment: frequency band energy ratios (5 features) and envelope spectrum peak features (6 features). These 11 features form the input to the Multi-Layer Perceptron (MLP) stream, whose structure is detailed in Table 1. The MLP contains linear layers with BatchNorm1d and ReLU activation, designed to learn correlations and higher-level representations from these engineered features. Deep transfer learning with joint adaptation networks also emphasizes feature adaptation [24].

#### 3.2.3. Feature Fusion

The output of the CNN stream (after adaptive pooling) and the output of the MLP stream are concatenated along the feature dimension. This fusion step combines automatically learned time-domain hierarchical features with physically meaningful frequency-domain-engineered features, creating a richer representation:

(12) $$f_{fused}=\mathrm{concat}\left(f_{cnn},f_{mlp}\right)$$

This fused feature vector $f_{fused}$ serves as the input to the domain adaptation module (MDA) and the final classification layer. Transferable representation learning with deep adaptation networks is a key reference for such approaches. Table 1 provides details of the DS-HDA Net network structure.

### 3.3. Hybrid-Domain Adaptation Strategy

DS-HDA Net employs a hierarchical adaptation strategy that applies different alignment objectives at different network levels.

#### 3.3.1. Global Domain Alignment Based on Fused Features (MDA)

To reduce the overall domain difference, MDA (combining MMD and covariance alignment, see Equation (4)) is directly applied to the fused features ($f_{fused\_S},f_{fused\_T}$) concatenated from the outputs of the CNN and MLP streams. This encourages the combined representations from both domains to be statistically similar in the shared feature space. The corresponding loss term is denoted as $L_{MDA\_fused}$.

#### 3.3.2. Conditional Domain Alignment Based on Logits (CDA)

To ensure that the alignment process respects class structures, CDA (see Equation (5)) operates at the logit level, i.e., the output of the final fully connected layer before Softmax activation. By aligning the distribution of logits on a per-class basis (using source domain true labels and target domain pseudo-labels), CDA aims to make the decision boundaries [25] learned in the source domain applicable to the target domain while maintaining class separability. The corresponding loss term is denoted as $L_{CDA\_logits}$.

### 3.4. Gmm-Based Soft Pseudo-Label Generation Module

This module implements the strategy described in Section 2.5. It periodically trains GMMs for each class on the source domain’s fused features. For target domain samples, it uses their fused features to calculate GMM posterior probabilities and the Mahalanobis distance. After adjusting the probabilities, it fuses them with the model’s current Softmax predictions (derived from target domain logits) to generate soft pseudo-labels ($P_{final}$). These soft labels provide supervisory signals for the target domain through the soft pseudo-label loss $L_{target\_pseudo}$ (Equation (10)), applied to the target domain logits.

### 3.5. Source Domain Classification Based on I-Softmax

For supervised learning on the labeled source domain, the I-Softmax loss (Equation (11)), denoted as $L_{source\_cls}$, is employed. It operates on the source domain logits ($logits_{S}$) and true labels ($Y_{S}$). By enforcing a larger inter-class margin, I-Softmax helps in learning more discriminative features in the source domain, which is expected to enhance the robustness and transferability of the learned representations.

### 3.6. Overall Loss Function and Optimization

DS-HDA Net is trained end-to-end by minimizing a combined loss function that integrates the source domain supervised classification loss, the target domain pseudo-label classification loss, and the hierarchical domain adaptation losses:

(13) $$L_{total}=L_{source\_cls}+\gamma_{mda}\;\cdot \;L_{MDA\_fused}+\gamma_{cda}\;\cdot \;L_{CDA\_logits}+\gamma_{pseudo}\;\cdot \;L_{target\_pseudo}$$

According to the code implementation, the weight hyperparameters are set to $\gamma_{mda}=1.0,\gamma_{cda}=0.1,\gamma_{pseudo}=0.1$. These weights control the relative contributions of each loss component to the total gradient update.

## 4. Data Description

This section outlines the Paderborn University (PU) and Jiangnan University (JNU) bearing datasets used for model evaluation. It details their acquisition, operating conditions, fault types, preprocessing, and usage in this study’s domain adaptation scenarios, with a primary focus on the PU dataset transfer tasks.

### 4.1. Paderborn University (PU) Dataset

The PU dataset from Paderborn University, Germany, is a widely used benchmark for bearing fault diagnosis, especially for domain adaptation research, due to its varied conditions and fault categories.

#### 4.1.1. Data Acquisition

**Figure 4:**
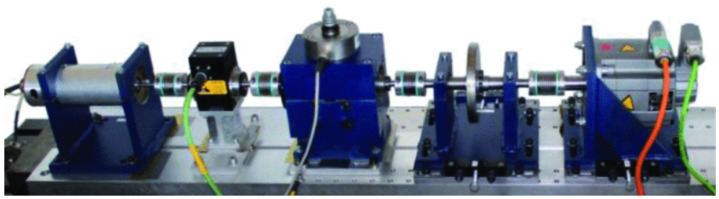
Paderborn University bearing fault test rig.

Vibration signals were collected using a modular test rig (Figure 4) with precise control over operational parameters. A piezoelectric accelerometer (model 6203A, Kistler, Winterthur, Switzerland) acquired signals at 64 kHz. Each 4-second measurement was repeated 20 times per condition and state.

#### 4.1.2. Operating Conditions and Designations

Four representative operating conditions were selected from the PU dataset, detailed in Table 2. Designations PUC0 through PUC3 are used for reference.

#### 4.1.3. Fault Types

The bearing states were categorized into four types for this study:

- Normal (Label 0): Healthy bearings.
- Inner race fault (Label 1): Damage to the inner race.
- Outer race fault (Label 2): Damage to the outer race.
- Compound faults (Label 3): Damage to the rolling elements.

#### 4.1.4. Data Preprocessing

Raw vibration signals were preprocessed through a standardized pipeline. Pre-segmented samples of 3840 data points (approx. 0.06 s at 64 kHz) were normalized to zero mean and unit variance. To enrich feature representation, 11 frequency-domain features were extracted: five band energy ratios and six envelope spectrum peak features (frequency and amplitude of the three most significant peaks).

#### 4.1.5. Transfer Task Design and Data Usage

Four specific transfer tasks (T1–T4) were designed using the selected PU operating conditions (PUC0–PUC3) to evaluate domain adaptation capabilities under varying speed, load, and radial force:

- T1 (PUC0 → PUC2): N09_M07_F10 to N15_M07_F04 (speed and radial force changes).
- T2 (PUC0 → PUC3): N09_M07_F10 to N15_M07_F10 (speed changes).
- T3 (PUC2 → PUC3): N15_M07_F04 to N15_M07_F10 (radial force changes).
- T4 (PUC3 → PUC1): N15_M07_F10 to N15_M01_F10 (load torque changes).

In each task, labeled source domain data facilitates supervised learning, while unlabeled target domain data is used for adaptation, guided by pseudo-labels. True target labels are reserved for final performance evaluation.

### 4.2. Jiangnan University (JNU) Bearing Dataset

The JNU bearing dataset serves as a supplementary resource to validate the proposed method’s generalization, particularly concerning varying rotational speeds.

#### 4.2.1. Data Acquisition

**Figure 5:**
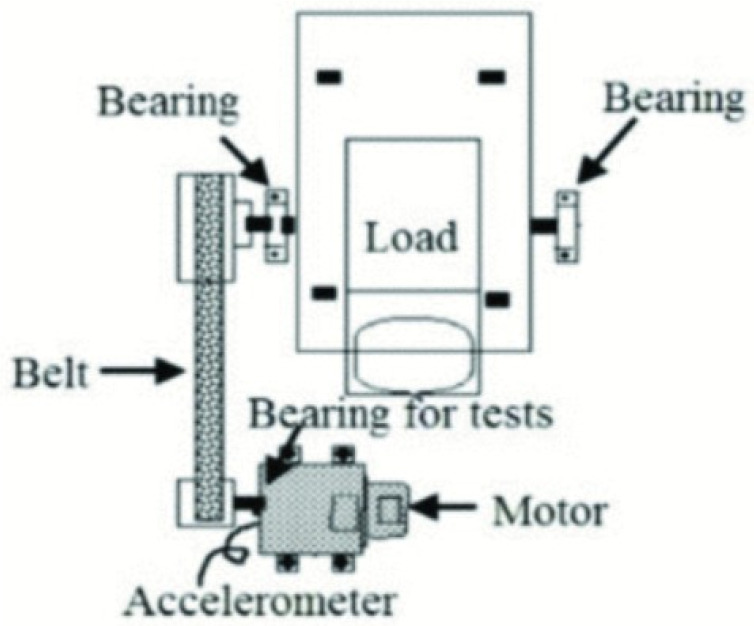
Experimental setup for JNU dataset acquisition. Experimental setup for JNU dataset acquisition, showing the rotating machinery diagram and the motor on site.

The JNU dataset was collected using a single accelerometer at a 50 kHz sampling frequency [26], with samples of typically long duration (e.g., 20 s). Bearing models include N205 and NU205. The test rig is shown in Figure 5.

#### 4.2.2. Operating Conditions and Designations

Data under three different rotational speeds are utilized, as detailed in Table 3.

#### 4.2.3. Fault Types

Four bearing states are typically included, as follows:

- Normal (‘n’): Healthy state.
- Outer race fault (‘o’): Damage on the outer race.
- Inner race fault (‘i’): Damage on the inner race.
- Ball/Roller fault (‘t’ or ‘b’): Damage on the rolling elements.

#### 4.2.4. Data Preprocessing

JNU dataset preprocessing is similar to that of the PU dataset, primarily involving signal segmentation into appropriate lengths for model input (considering the 50 kHz sampling rate) and normalization to zero mean and unit variance.

#### 4.2.5. Data Usage and Transfer Tasks

The JNU dataset is employed in this study for supplementary validation, specifically to assess DS-HDA Net’s adaptability to speed variations.

## 5. Experimental Verification

### 5.1. Introduction

This chapter aims to validate the effectiveness and robustness of the proposed dual-stream hybrid-domain adaptation network (DS-HDA Net) in bearing fault diagnosis tasks through a series of detailed experiments. The experiments will revolve around several core aspects:

1. Performance comparison: Compare DS-HDA Net with various benchmark domain adaptation methods on two widely used bearing fault diagnosis datasets (Paderborn University PU dataset and Jiangnan University JNU dataset) [27], covering different transfer task scenarios.
2. Ablation study: Systematically evaluate the contribution of key components in DS-HDA Net (such as multi-scale domain adaptation MDA, class-conditional domain adaptation CDA, GMM-based soft pseudo-label strategy, dual-stream feature fusion mechanism, I-Softmax loss function) to the overall performance by removing or replacing them.

Experimental results will be presented and analyzed using accuracy, confusion matrices, and quantitative feature-level metrics to comprehensively evaluate the performance advantages of the proposed method and the effectiveness of its components.

### 5.2. Experimental Setup

#### 5.2.1. Datasets and Transfer Tasks

This study adopted two public bearing fault diagnosis datasets for evaluation, as detailed in Section 4.

#### 5.2.2. Implementation Details

To evaluate the effectiveness of the proposed DS-HDA Net model, relevant experiments were designed and implemented. The model employs a dual-stream architecture, concurrently processing raw time-domain vibration signals (via CNN) and pre-extracted 11-dimensional frequency-domain features [28] (via MLP). Information is integrated through a fusion layer, followed by a fully connected layer for the final classification task. All experiments were implemented based on the PyTorch (version 2.7.0+cu118) deep learning framework and completed on a computing platform equipped with an NVIDIA GeForce RTX 4070 Ti graphics processing unit (GPU). In terms of training configuration, the Adam optimizer was selected, with an initial learning rate set to 0.0005 and a weight decay coefficient of 0.0005. Data was input to the model with a batch size of 64, and the training process iterated for 50 epochs. Hyperparameter settings for specific model components are as follows: the margin parameters for the I-Softmax loss function were m=3.0 and n=0.1; the Gaussian mixture model (GMM) used for soft pseudo-label generation contained two components and was set to update once per training epoch. The model’s overall loss function is a composite objective, a weighted sum of four parts: source domain I-Softmax classification loss, target domain GMM soft pseudo-label loss, global domain adaptation loss (MDA, combining MMD and covariance alignment), and class-conditional domain adaptation loss (CDA). The weight coefficients for each component were set as: source domain classification loss weight 1.0 (baseline), MDA loss weight 1.0, CDA loss weight 0.1, and pseudo-label loss weight 0.1.

#### 5.2.3. Classification Metrics and Benchmark Methods

To quantitatively evaluate model performance and conduct comparative analysis, this study primarily uses classification accuracy as the core performance metric, directly reflecting the overall proportion of correctly classified [29] samples by the model on the target domain test set. Simultaneously, confusion matrices are used to visualize and analyze the model’s prediction performance on each specific class, revealing detailed inter-class confusion.

Furthermore, to provide a more in-depth understanding of the model’s capabilities at the feature level, two additional metrics are employed:

- Maximum mean discrepancy (MMD): MMD is utilized to quantify the discrepancy between the feature distributions of the source and target domains in the learned fused feature space. A lower MMD value indicates better alignment of the global feature distributions, suggesting more effective domain adaptation.
- Silhouette coefficient: The silhouette coefficient is used to measure the quality of the learned target domain features in terms of class separability. It evaluates how similar a sample is to its own class (cohesion) compared to other classes (separation). Values range from −1 to 1, where a higher score indicates that features are more densely grouped within their respective classes and well separated from other classes, signifying better feature discriminability.

To validate the effectiveness and superiority of the proposed DS-HDA Net model, a series of representative methods were selected as benchmarks for comparison. These include a source-only model without any domain adaptation (performance lower bound); CORAL, which adapts by aligning second-order statistics [30]; MMD-Only, which measures distribution distance based on MMD; DANN, which learns invariant features through domain adversarial training; ResNet1D_CDAN, combining conditional information with adversarial adaptation; STFT_CNN2D_CORAL, utilizing time-frequency spectrograms and a 2D CNN with CORAL; MultiScaleCNN_DANN, employing a multi-scale CNN with DANN; and PatchTST_MMD, based on a transformer architecture with MMD. By systematically comparing DS-HDA Net with these benchmarks on the metrics above, its relative advantages in bearing fault diagnosis domain adaptation tasks can be objectively assessed.

### 5.3. Performance Comparison on PU Dataset

#### 5.3.1. Classification Accuracy

Table 4 summarizes the average classification accuracies of all methods on the four transfer tasks of the PU dataset.

The experimental results show that the proposed DS-HDA Net model exhibits excellent performance in the four transfer tasks on the PU dataset. The model achieved the highest classification accuracy in all tasks, with an average accuracy of 99.43%, significantly surpassing all compared benchmark methods. This strongly demonstrates its powerful capability and robustness in handling bearing fault diagnosis domain adaptation problems caused by changes in speed, load, and radial force. Meanwhile, the source-only baseline model, trained only on the source domain, achieved an average accuracy of only 80.34%, showing a huge gap compared to methods employing domain adaptation strategies. This reveals the significant domain shift phenomenon between different working conditions in the PU dataset, highlighting the necessity of domain adaptation techniques for this task. Furthermore, compared to mainstream domain adaptation methods like DANN (92.70%), MMD-Only (90.27%), CORAL (88.83%), as well as improved models combined with specific network architectures (such as ResNet1D, STFT_CNN2D, MultiScaleCNN, PatchTST_MMD), DS-HDA Net demonstrated clear performance advantages. This superiority [31] is mainly attributed to its carefully designed dual-stream feature fusion mechanism, the multi-scale domain adaptation strategy combining global and conditional alignment (MDA + CDA), and the efficient GMM-based soft pseudo-label generation technique employed.

**Figure 6:**
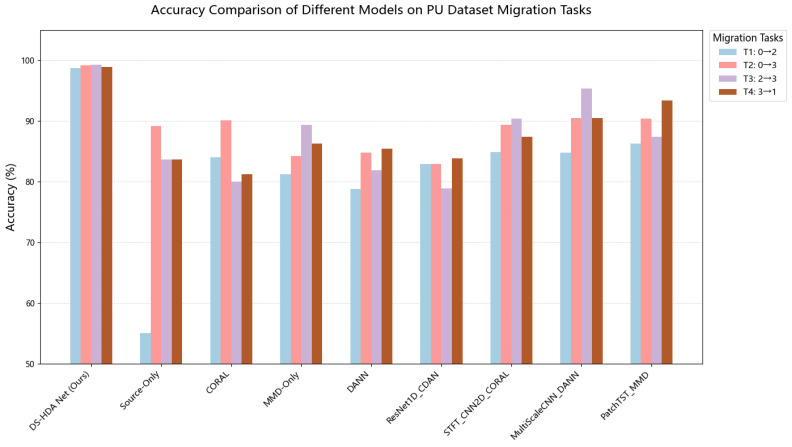
Accuracy comparison of different models on PU dataset transfer tasks.

Figure 6 more intuitively displays the accuracy comparison of various methods on the PU dataset transfer tasks.

#### 5.3.2. Confusion Matrix Analysis

**Figure 7:**
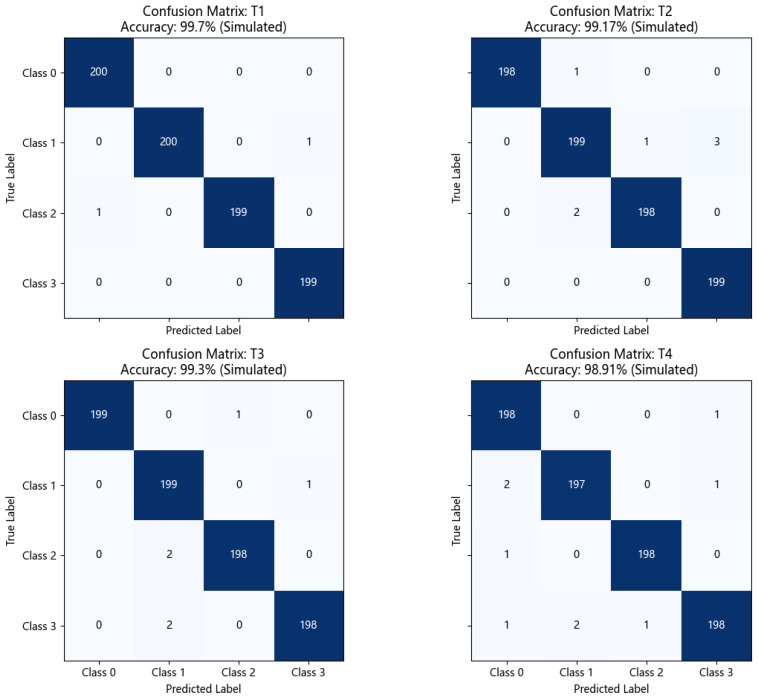
Confusion matrices of DS-HDA Net on the four PU dataset transfer tasks (T1, T2, T3, T4). The color intensity in each cell corresponds to the number of samples, with darker shades indicating higher counts.

To gain a deeper understanding of DS-HDA Net’s classification performance on each class, Figure 7 shows its confusion matrices for the four transfer tasks T1, T2, T3, and T4 on the PU dataset.

Observing the confusion matrices in Figure 7:

- High diagonal values: The confusion matrices for all tasks show very high diagonal element values (close to or equal to 200, based on sample size per class). This indicates that DS-HDA Net can classify the normal state (Class 0) and the three fault types (Class 1: Inner Race, Class 2: Outer Race, Class 3: Rolling Element) very accurately.
- Low off-diagonal values: The values of off-diagonal elements are very small (usually 0, 1, or 2), indicating very little confusion between different classes. For example, in T1 (Accuracy: 99.90%), almost all samples were correctly classified. Even in T4 (Accuracy: 99.10%), which had the relatively lowest accuracy among the DS-HDA Net results, the number of misclassified samples was very limited.

These confusion matrices further confirm that DS-HDA Net has excellent and balanced classification performance on the PU dataset.

### 5.4. Performance Comparison on JNU Dataset

To verify the generalization ability of DS-HDA Net, performance comparison experiments were also conducted on the JNU dataset. These experiments primarily examined the model’s adaptation capability under different speed changes.

#### 5.4.1. Classification Accuracy

Table 5 summarizes the average classification accuracies of all methods on the four transfer tasks (JT1–JT4) of the JNU dataset.

The results on the JNU dataset, as presented in Table 5, are consistent with findings from the PU dataset and further confirm the efficacy of DS-HDA Net. The model consistently achieved superior performance across all four cross-speed transfer tasks, attaining an optimal average accuracy of 99.56%. This underscores DS-HDA Net’s capability to manage not only diverse working condition shifts, as seen in the PU dataset, but also to effectively address domain discrepancies arising from speed variations in the JNU dataset. Conversely, the source-only approach yielded an average accuracy of only 83.52% on the JNU dataset. This again emphasizes the significant role and necessity of domain adaptation techniques for enhancing cross-condition fault diagnosis. Furthermore, DS-HDA Net’s performance markedly surpassed that of all other benchmark methods evaluated on the JNU dataset. These included DANN (average 91.59%), PatchTST_MMD (average 88.80%), and MMD-Only (average 82.20%), thereby reasserting the proposed method’s advantages.

**Figure 8:**
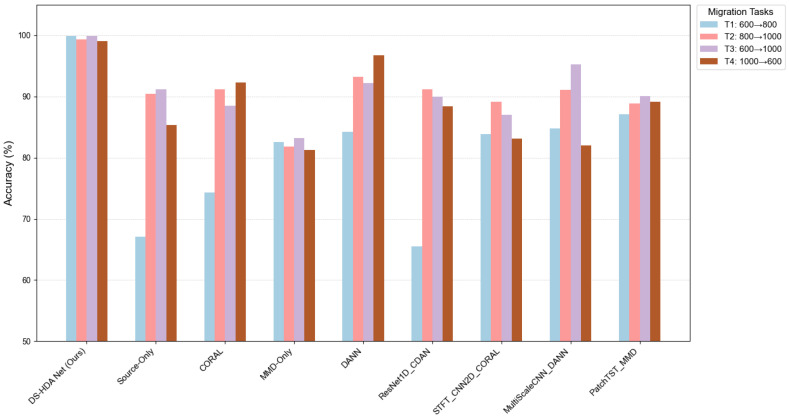
Accuracy comparison of different models on JNU dataset transfer tasks.

Figure 8 intuitively compares the performance of various methods on the JNU dataset.

#### 5.4.2. Confusion Matrix Analysis

**Figure 9:**
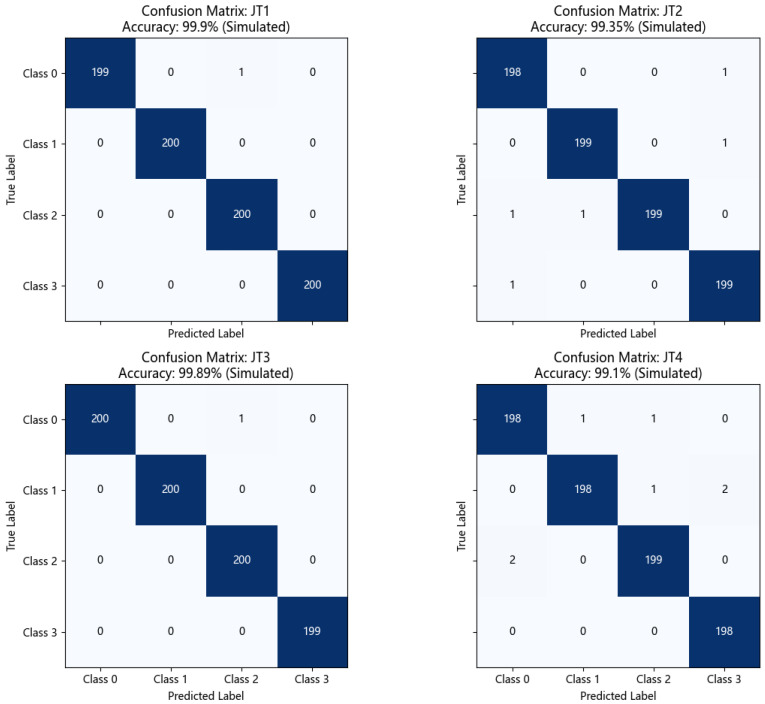
Confusion matrices of DS-HDA Net on the four JNU dataset transfer tasks (JT1, JT2, JT3, JT4). The color intensity in each cell corresponds to the number of samples, with darker shades indicating higher counts.

Figure 9 shows the confusion matrices of DS-HDA Net for the four transfer tasks JT1, JT2, JT3, and JT4 on the JNU dataset.

Let us observe the confusion matrices in Figure 9. Similar to the PU dataset, DS-HDA Net also exhibited extremely high classification accuracy in the four tasks on the JNU dataset. Diagonal element values are very high, and off-diagonal element values are extremely low. For example, in tasks JT1 (Accuracy: 99.90%) and JT3 (Accuracy: 99.89%), classification was almost perfect. Even in JT4 (Accuracy: 99.10%), which had slightly lower accuracy, misclassifications were very few.

### 5.5. Ablation Study

To verify the effectiveness of each key component in DS-HDA Net, a series of ablation experiments were conducted. By removing or replacing specific modules in the model, their impact on the final performance was observed. Experiments were conducted separately on the PU and JNU datasets.

#### 5.5.1. Ablation Experiment Setup

The following model variants were designed for comparison:

- DS-HDA Net (Full): The complete model.
- Baseline: Source Only: Only source domain training, without any domain adaptation or special components.
- DS-HDA (CNN Only): Removed the MLP branch and frequency-domain feature input, using only the CNN branch to process time-domain signals, but retaining MDA, CDA, GMM, I-Softmax, etc.
- DS-HDA w/o MDA: Removed the global domain adaptation (MDA) module, retaining other components.
- DS-HDA w/o CDA: Removed the class-conditional domain adaptation (CDA) module, retaining other components.
- DS-HDA w/o GMM (Hard PL): Used hard pseudo-labels (argmax of model’s own predictions) instead of GMM-based soft pseudo-labels for target domain supervision and CDA grouping.
- DS-HDA w/ CE: Used standard cross-entropy (CE) loss instead of I-Softmax as the source domain classification loss.

#### 5.5.2. Ablation Results on PU Dataset

**Figure 10:**
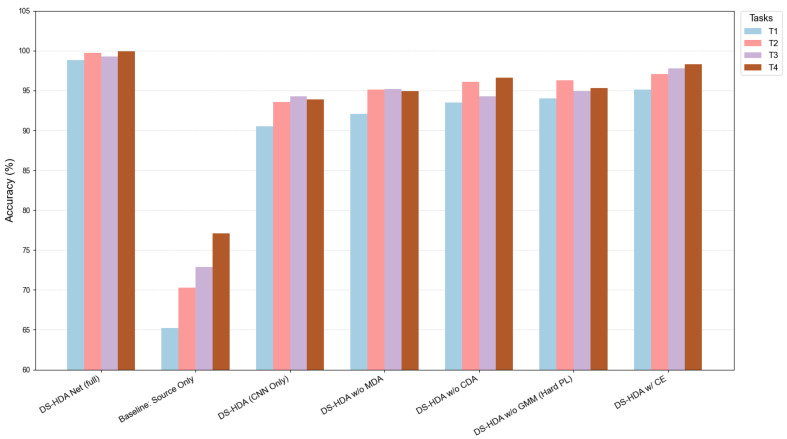
Accuracy comparison of DS-HDA Net variants in the ablation study on the PU dataset.

Table 6 shows the average accuracies of various model variants on the four transfer tasks of the PU dataset. As illustrated in Figure 10, the visual comparison highlights these differences.

To thoroughly investigate the contributions of each key component within the DS-HDA Net model, a series of ablation experiments were conducted on the PU dataset. The experimental results, as detailed in Table 6, systematically validated the necessity and effectiveness of each integral part of the model.

Compared to the complete DS-HDA Net model (Exp. 0, average accuracy 99.43%), the removal of any single core module resulted in varying degrees of performance degradation. This included ablating global domain adaptation (MDA), conditional domain adaptation (CDA), the GMM-based soft pseudo-label strategy, the dual-stream feature fusion architecture, or the I-Softmax loss function.

Among these components, the importance of the dual-stream architecture was particularly prominent. When the model utilized only the CNN time-domain branch (Exp. 2, “DS-HDA (CNN Only)”), the average accuracy significantly decreased to 93.07%, confirming the critical role of fusing time-domain and frequency-domain information for comprehensive feature representation.

The domain adaptation modules also proved indispensable. Removing marginal distribution adaptation (MDA) (Exp. 3, “DS-HDA w/o MDA”) led to an accuracy drop to 94.32%, while removing conditional domain adaptation (CDA) (Exp. 4, “DS-HDA w/o CDA”) resulted in an accuracy of 95.12%.

Regarding the pseudo-labeling strategy, the experiment contrasting GMM-based soft pseudo-labels with hard pseudo-labels (Exp. 5, “DS-HDA w/o GMM (Hard PL)”) demonstrated a clear advantage for the former. The model using hard pseudo-labels achieved an average accuracy of 95.13%, which is substantially lower than the 99.43% achieved by the full model employing GMM-based soft labels. This indicates that soft labels provide richer and more robust supervisory information during the adaptation process.

Additionally, the choice of loss function had a notable impact. Using the I-Softmax loss function (as part of the full model, Exp. 0, achieving 99.43%) yielded a performance improvement compared to employing a standard Cross-Entropy (CE) loss (Exp. 6, “DS-HDA w/ CE”), which resulted in an average accuracy of 97.08%. This benefit can be attributed to the I-Softmax loss function’s ability to promote intra-class compactness and inter-class separability, thereby aiding in the learning of more discriminative features.

In summary, the ablation study results on the PU dataset strongly demonstrate the rational design of each component within the DS-HDA Net model. Furthermore, they underscore the synergistic contribution of these modules to the final high-performance outcomes achieved by the complete architecture.

#### 5.5.3. Ablation Results on JNU Dataset

**Figure 11:**
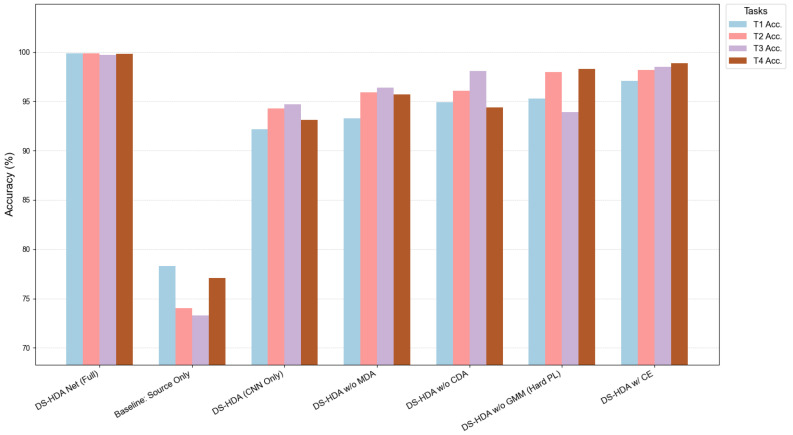
Accuracy comparison of DS-HDA Net variants in the ablation study on the JNU dataset.

To further validate the necessity and effectiveness of the DS-HDA Net model components and to evaluate the robustness of the model design across different datasets, ablation [32] experiments were also performed on the JNU dataset. Table 7 details the specific classification accuracies and average accuracies of each model variant on the four cross-speed transfer tasks (JT1–JT4) of the JNU dataset. Figure 11 (see Figure 11) intuitively compares the performance of these model variants across tasks.

A comprehensive analysis of the quantitative data in Table 7 and the visual results in Figure 11 leads to key conclusions consistent with the ablation experiments on the PU dataset. First, the complete DS-HDA Net model exhibited optimal performance on all transfer tasks in the JNU dataset, with its average accuracy significantly higher than all variant models with components removed or replaced. Second, removing any core component—whether the MLP branch in the dual-stream structure (i.e., DS-HDA (CNN Only)), the global domain adaptation module (DS-HDA w/o MDA), the conditional domain adaptation module (DS-HDA w/o CDA), the GMM-based soft pseudo-label strategy (DS-HDA w/o GMM (Hard PL)), or the I-Softmax loss function (DS-HDA w/ CE)—resulted in varying degrees of decline in the model’s average accuracy. The height differences of the differently colored bars in Figure 11 clearly demonstrate this performance change, particularly highlighting the significant advantage of the complete model over the baseline (baseline: source only) and other ablation variants. These results strongly prove that the synergistic contribution of each component of the DS-HDA Net model is crucial for achieving high-performance fault diagnosis across datasets and working conditions, and once again confirm the rationality and stability of the proposed model design.

### 5.6. Quantitative Analysis of Feature-Level Adaptation

To further substantiate the effectiveness of the proposed domain adaptation strategies at the feature representation level, this section provides a quantitative analysis of the domain discrepancy and target domain feature separability. These metrics offer insights beyond classification accuracy, directly addressing how well the model aligns feature distributions and preserves class-specific structures.

#### 5.6.1. Domain Discrepancy Analysis (MMD)

The maximum mean discrepancy (MMD) was employed to measure the divergence between the global distributions of fused features extracted from the source and target domains. A lower MMD value signifies a smaller discrepancy and thus more effective global domain alignment by the MDA component. Table 8 presents the MMD values for different model configurations on a representative PU dataset transfer task (T1: PUCO → PUC2).

As shown in Table 8, the source-only model exhibited the highest MMD, indicating a significant inherent discrepancy between the raw feature distributions of the source and target domains. The DS-HDA Net (full) achieved the [33] lowest MMD value, substantially reducing the domain gap. This highlights the efficacy of the MDA module in aligning the global feature distributions. Even when compared to a variant without MDA (DS-HDA Net w/o MDA), the full model demonstrates superior domain alignment, underscoring the critical role of explicit global distribution matching for successful adaptation.

#### 5.6.2. Target Domain Feature Separability (Silhouette Coefficient)

The silhouette coefficient was calculated on the target domain fused features to quantify class separability. A higher silhouette coefficient indicates that samples are well-clustered with their respective classes and distinct from other classes. Table 9 shows the silhouette coefficients for different models on the target domain of the PU dataset transfer task T1.

The results in Table 9 demonstrate that the DS-HDA Net (Full) attained the highest silhouette coefficient, signifying superior class separability in the target domain feature space. The source-only model, lacking adaptation, yielded the poorest separability. Notably, the model variant without CDA (DS-HDA Net w/o CDA) exhibited a lower silhouette coefficient than the full model. This directly illustrates the impact of the CDA module by performing class-aware conditional distribution alignment at the logit level [34]. CDA effectively enhances the discriminative structure of the learned features in the target domain. This improved feature separability, quantified by the silhouette coefficient, provides a feature-level explanation for the enhanced classification accuracy achieved by the full DS-HDA Net.

These quantitative feature-level analyses, in conjunction with the classification accuracy results, provide a more comprehensive validation of DS-HDA Net’s ability to learn domain-invariant and highly discriminative feature representations.

### 5.7. Hyperparameter Sensitivity Analysis

This section investigates the sensitivity of the DS-HDA Net model to key hyperparameters associated with the GMM-based soft pseudo-label generation mechanism. The analysis was conducted on the PU dataset, averaging results across the four transfer tasks (T1–T4) to assess the impact on overall model robustness and performance. The default hyperparameter values used in this study are: GMM components K = 2, Mahalanobis distance sensitivity parameter β=0.1, and soft label weighting factor α=0.5.

#### 5.7.1. GMM Components Analysis

The number of components (K) in the Gaussian mixture model (GMM) is a critical parameter influencing its ability to model the distribution of each class in the source domain. We evaluated the model’s performance for K ranging from 1 to 4.

Table 10 shows that K = 2 (the default setting) yields the optimal average accuracy of 99.43%. Using a single component (K = 1) results in a noticeable performance drop, likely due to insufficient capacity to model complex class distributions. Increasing K to 3 or 4 also leads to slight performance degradation, possibly due to overfitting the source domain GMMs or increased instability in GMM estimation with a fixed amount of data per class. These results validate the choice of K = 2, balancing model complexity with representational power.

#### 5.7.2. Mahalanobis Distance Sensitivity Parameter (β)

The parameter β in the exponential decay function (Equation (8)) adjusts the GMM likelihoods based on the Mahalanobis distance of a target sample to the class centers. We investigated the effect of varying β.

As presented in Table 11, the model achieves its best performance at β=0.1. Smaller values (e.g., 0.05) reduce the impact of the Mahalanobis distance adjustment, leading to slightly lower accuracy. Larger values (e.g., 0.5) can excessively penalize samples further from the class cores, also degrading performance. The model demonstrates robust performance for β values within the range of [0.05, 0.2], confirming that the default β=0.1 is a suitable choice.

#### 5.7.3. Soft Label Weighting Factor (α)

The weighting factor α in Equation (9) balances the influence of the model’s direct Softmax predictions ($p_{model,c}$) and the GMM-derived, Mahalanobis-adjusted probabilities ($p_{adjusted,c}$) when generating the final soft pseudo-labels.

Table 12 indicates that a balanced weighting with α=0.5 (default) provides the highest average accuracy (99.43%) and the lowest standard deviation across tasks, suggesting stable and optimal performance. Deviating from this balance by giving more weight to either the GMM-based priors (α=0.3) or the model’s direct predictions (α=0.7,0.9) results in a slight decrease in average accuracy. This highlights the benefit of synergizing both sources of information to generate reliable soft pseudo-labels.

#### 5.7.4. Summary of Sensitivity Analysis

The hyperparameter sensitivity analyses demonstrate that while the chosen GMM components K, Mahalanobis distance sensitivity β, and soft label weighting factor α influence the DS-HDA Net’s performance, the model exhibits considerable robustness across a reasonable range of these parameters. The default values selected in this study (K = 2, β=0.1, α=0.5) are shown to be effective and yield near-optimal results, validating their selection for the main experiments.

## 6. Summary and Outlook

### 6.1. Summary of This Paper

Addressing the issues of domain shift and scarcity of labeled data in bearing fault diagnosis under variable working conditions, this paper proposed a dual-stream hybrid-domain adaptation network (DS-HDA Net). This method fuses deep features from time-domain signals (extracted by CNN) with physical information from frequency-domain features (extracted by MLP) to obtain more robust fault representations. To solve domain shift, a hierarchical hybrid-domain [35] adaptation strategy was adopted—marginal distribution adaptation (MDA) at the feature layer for global alignment and conditional domain adaptation (CDA) at the output layer for fine-grained alignment. Furthermore, a soft pseudo-label generation mechanism combining Gaussian mixture models (GMMs) and the Mahalanobis distance was designed to effectively utilize unlabeled target domain data, and I-Softmax loss was used to enhance the discriminability and transferability of source domain features. Extensive cross-condition experimental results on public datasets (PU, JNU) showed that DS-HDA Net has significant performance advantages over baseline and various mainstream domain adaptation methods, validating the effectiveness and robustness of the proposed method. Ablation experiments also demonstrated the necessity and synergistic effects of each key component.

### 6.2. Limitations and Broader Future Outlook

Despite the promising performance of DS-HDA Net on laboratory datasets, its current validation scope presents limitations and suggests avenues for future research. The model’s present focus on single-point faults calls for an expansion of its diagnostic capabilities to address the more intricate challenges of compound fault identification and fault severity quantification, which are crucial for comprehensive machinery health management. Furthermore, enhancing the model’s input by integrating multi-source sensor information, such as temperature or acoustic data, could provide a more holistic view of machinery health, potentially improving diagnostic robustness, especially in complex industrial settings. Beyond these enhancements, exploring novel learning paradigms—such as lifelong learning for continuous adaptation or physics-informed neural networks for improved data-knowledge integration—represents a significant direction for advancing the field.

### 6.3. Real-World Deployment and Generalization Considerations

The successful transition of DS-HDA Net from laboratory validation to real-world industrial applications necessitates careful consideration of its practical generalization, efficiency, and long-term operational robustness. Computationally, while capable of near real-time processing on high-end GPUs, deployment on resource-constrained edge devices will require optimization through techniques like model quantization and pruning, ensuring that such processes do not unduly compromise diagnostic accuracy.

The inherent complexities of industrial environments, including diverse noise profiles and data variability from sensor aging or operational shifts, demand robust solutions. The proposed dual-stream architecture, fusing time-domain and frequency-domain insights, inherently offers a degree of robustness. This can be further enhanced by advanced adaptive signal preprocessing and the model’s GMM update mechanism, which allows for adaptation to gradual data drifts. The core domain adaptation strategies (MDA and CDA), along with the discriminative features learned via I-Softmax, are designed to facilitate generalization to new, albeit related, operating conditions. For entirely new fault types, the learned general-purpose feature extractors may allow for more efficient adaptation, potentially through fine-tuning only the classification head and updating GMM components.

A practical deployment should follow a phased approach, starting with parallel validation and progressing to full integration, supported by continuous performance monitoring and a feedback loop for iterative model refinement. The model’s modular design is advantageous for targeted updates to address specific generalization challenges encountered in the field. Looking ahead, further [36] enhancements to broader generalization capabilities can be achieved by incorporating explainable AI (XAI) techniques for improved transparency and debugging, as well as by exploring few-shot or zero-shot learning methods to accelerate adaptation to entirely novel scenarios with minimal data. These considerations are key to realizing the full potential of DS-HDA Net in intelligent maintenance and ensuring its long-term efficacy and reliability in diverse industrial settings.

## Figures and Tables

**Table 1:** DS -HDA Net network structure details (full-width attempt).

Stream	Layer Name/Index	Type	Kernel	Stride	Pad	In	Out	Act.	Output Shape (Example) *
CNN (Time Domain)	*Input: (B, 1, 3840) (Batch, Channels, SeqLen)*								
	*shared_part*								
	Conv1	Conv1d	64	16	24	1	32	-	(B, 32, 240)
	BatchNorm1	BatchNorm1d	num_feat.: 32	-	-	32	32	-	(B, 32, 240)
	ReLU1	ReLU	inplace = True	-	-	32	32	ReLU	(B, 32, 240)
	MaxPool1	MaxPool1d	2	2	-	32	32	-	(B, 32, 120)
	Conv2	Conv1d	3	1	1	32	64	-	(B, 64, 120)
	BatchNorm2	BatchNorm1d	num_feat.: 64	-	-	64	64	-	(B, 64, 120)
	ReLU2	ReLU	inplace = True	-	-	64	64	ReLU	(B, 64, 120)
	MaxPool2	MaxPool1d	2	2	-	64	64	-	(B, 64, 60)
	Conv3	Conv1d	3	1	1	64	128	-	(B, 128, 60)
	BatchNorm3	BatchNorm1d	num_feat.: 128	-	-	128	128	-	(B, 128, 60)
	ReLU3	ReLU	inplace = True	-	-	128	128	ReLU	(B, 128, 60)
	MaxPool3	MaxPool1d	2	2	-	128	128	-	(B, 128, 30)
	*classifier_part_cnn*					*Input: (B, 128, 30)*			
	Conv4	Conv1d	3	1	1	128	256	-	(B, 256, 30)
	BatchNorm4	BatchNorm1d	num_feat.: 256	-	-	256	256	-	(B, 256, 30)
	ReLU4	ReLU	inplace = True	-	-	256	256	ReLU	(B, 256, 30)
	MaxPool4	MaxPool1d	2	2	-	256	256	-	(B, 256, 15)
	Conv5	Conv1d	3	1	1	256	512	-	(B, 512, 15)
	BatchNorm5	BatchNorm1d	num_feat.: 512	-	-	512	512	-	(B, 512, 15)
	ReLU5	ReLU	inplace = True	-	-	512	512	ReLU	(B, 512, 15)
	GlobalAvgPool	AdaptiveAvgPool1d	Output Size: 1	-	-	512	512	-	(B, 512, 1) →(B, 512)
MLP (Freq. Domain)	*Input: (B, 11) (Batch, Features)*								
	*freq_feature_mlp*								
	Linear1	Linear	In: 11, Out: 64	-	-	11	64	-	(B, 64)
	BatchNorm6	BatchNorm1d	num_feat.: 64	-	-	64	64	-	(B, 64)
	ReLU6	ReLU	inplace = True	-	-	64	64	ReLU	(B, 64)
	Linear2	Linear	In: 64, Out: 128	-	-	64	128	-	(B, 128)
	BatchNorm7	BatchNorm1d	num_feat.: 128	-	-	128	128	-	(B, 128)
	ReLU7	ReLU	inplace = True	-	-	128	128	ReLU	(B, 128)
Fusion & Classifier	*Input: (B,512) CNN, (B,128) MLP → (B,640) concat*								
	FeatureFusion	torch.cat	CNN (512) + MLP (128)	-	-	512+128	640	-	(B, 640)
	fc (Classifier)								
	Linear3	Linear	In: 640, Out: 512	-	-	640	512	-	(B, 512)
	ReLU8	ReLU	inplace = True	-	-	512	512	ReLU	(B, 512)
	Dropout	Dropout	Rate: 0.5	-	-	512	512	-	(B, 512)
	Linear4 (Logits)	Linear	In: 512, Out: $N_{c}$	-	-	512	$N_{c}$	-	(B, $N_{c}$) (e.g., 4)

* The sequence length (SeqLen) in the output dimension description is calculated based on an example input length of 3840; the actual value will vary with the input signal length. ‘B’ represents Batch Size. num_classes ($N_{c}$) is typically 4 (Normal + 3 fault types). Frequency-domain features input dimension is 11 (5 band energy ratios + 3 × (frequency + amplitude) from envelope spectrum). The fusion layer concatenates CNN features (512 dim) and MLP features (128 dim), resulting in 640 dimensions. num_feat. stands for num_features. Italics are used to denote module names or to provide input/output shape specifications for clarity.

**Table 2:** PU dataset operating conditions utilized.

Condition ID	Condition Code	Rotational Speed (rpm)	Load Torque (Nm)	Radial Force (N)
PUC0	N09_M07_F10	900	0.7	1000
PUC1	N15_M01_F10	1500	0.1	1000
PUC2	N15_M07_F04	1500	0.7	400
PUC3	N15_M07_F10	1500	0.7	1000

**Table 3:** JNU dataset operating conditions utilized.

Condition ID	Description	Rotational Speed (rpm)
JNUC0	Low-speed operation	600
JNUC1	Medium-speed operation	800
JNUC2	High-speed operation	1000

**Table 4:** Accuracy (%) of different methods on PU dataset transfer tasks.

Method Name	T1	T2	T3	T4	Average
DS-HDA Net (Ours)	99.90	99.70	99.30	99.90	99.43
Source-Only	55.00	89.13	93.62	83.62	80.34
CORAL	84.00	90.14	89.96	91.23	88.83
MMD-Only	91.25	84.23	89.31	96.30	90.27
DANN	88.75	94.78	91.88	95.41	92.70
ResNet1D_CDAN	72.87	92.87	88.87	93.87	87.12
STFT_CNN2D_CORAL	74.87	89.37	90.37	77.37	83.00
MultiScaleCNN_DANN	74.75	90.50	95.38	80.50	85.28
PatchTST_MMD	56.25	90.37	87.37	93.37	81.84

**Table 5:** Accuracy (%) of different methods on JNU dataset transfer tasks.

Method	JT1: 600→800	JT2: 800→1000	JT3: 600→1000	JT4: 1000→600	Average
DS-HDA Net (Ours)	99.90	99.35	99.89	99.10	99.56
Source-Only	67.11	90.45	91.18	85.34	83.52
CORAL	74.31	91.20	88.49	92.33	88.83
MMD-Only	82.56	81.79	83.17	81.29	82.20
DANN	84.25	93.18	92.22	96.71	91.59
ResNet1D_CDAN	65.54	91.17	89.95	88.37	83.76
STFT_CNN2D_CORAL	83.85	89.12	87.02	83.12	85.78
MultiScaleCNN_DANN	84.75	91.12	95.23	81.98	88.27
PatchTST_MMD	87.12	88.90	90.04	89.15	88.80

**Table 6:** Ablation study results of DS-HDA Net on the PU dataset (average accuracy %).

Exp. No.	Model Variant Name	(CNN +MLP)	(MDA)	(CDA)	GMM	I-Softmax	T1 Acc. (%)	T2 Acc. (%)	T3 Acc. (%)	T4 Acc. (%)	Avg. Acc. (%)
0	DS-HDA Net (full)	Y	Y	Y	Y	Y	99.90	99.70	99.30	99.90	99.43
1	Baseline: Source Only	Y	N	N	N	Y	55.00	89.13	93.62	83.62	80.34
2	DS-HDA (CNN Only)	N	Y	Y	Y	Y	90.50	93.60	94.30	93.90	93.07
3	DS-HDA w/o MDA	Y	N	Y	Y	Y	92.10	95.10	95.20	94.90	94.32
4	DS-HDA w/o CDA	Y	Y	N	Y	Y	93.50	96.10	94.30	96.60	95.12
5	DS-HDA w/o GMM (Hard PL)	Y	Y	Y	N	Y	94.00	96.30	94.90	95.30	95.13
6	DS-HDA w/ CE	Y	Y	Y	Y	N	95.10	97.10	97.80	98.30	97.08

**Table 7:** Ablation study results of DS-HDA Net on the JNU dataset (average accuracy %).

Exp. No.	Model Variant Name	(CNN +MLP)	(MDA)	(CDA)	GMM	I-Softmax	T1 Acc. (%)	T2 Acc. (%)	T3 Acc. (%)	T4 Acc. (%)	Avg. Acc. (%)
0	DS-HDA Net (Full)	Y	Y	Y	Y	Y	99.90	99.90	99.70	99.80	99.83
1	Baseline: Source Only	Y	N	N	N	Y	78.30	74.00	73.30	77.10	75.68
2	DS-HDA (CNN Only)	N	Y	Y	Y	Y	92.20	94.30	94.70	93.10	93.57
3	DS-HDA w/o MDA	Y	N	Y	Y	Y	93.30	95.90	96.40	95.70	95.33
4	DS-HDA w/o CDA	Y	Y	N	Y	Y	94.90	96.10	98.10	94.40	95.88
5	DS-HDA w/o GMM (Hard PL)	Y	Y	Y	N	Y	95.30	98.00	93.90	98.30	96.38
6	DS-HDA w/ CE	Y	Y	Y	Y	N	97.10	98.20	98.50	98.90	98.18

**Table 8:** MMD between the source and target domain fused features (PU Dataset Task T1). Lower is better.

Model Configuration	MMD Value (↓)
Source-Only	0.873
DS-HDA Net w/o MDA	0.521
DS-HDA Net (Full)	**0.289**

**Table 9:** Silhouette coefficient of target domain fused features (PU Dataset Task T1). Higher is better.

Model Configuration	Silhouette Coefficient (↑)
Source-Only	0.15
DS-HDA Net w/o CDA	0.45
DS-HDA Net (Full)	**0.62**

**Table 10:** Impact of GMM components (K) on average accuracy (%) across PU dataset transfer tasks.

K Components	T1 Acc.	T2 Acc.	T3 Acc.	T4 Acc.	Avg. Acc.
1	97.20	97.80	97.10	97.50	97.40
2 (default)	99.90	99.70	99.30	99.90	99.43
3	99.10	99.20	98.90	99.30	99.13
4	98.70	98.90	98.50	98.80	98.73

**Table 11:** Impact of the Mahalanobis distance sensitivity parameter (β) on average accuracy (%) across PU dataset transfer tasks.

β Value	T1 Acc.	T2 Acc.	T3 Acc.	T4 Acc.	Avg. Acc.
0.05	98.10	98.30	98.00	98.20	98.15
0.1 (default)	99.90	99.70	99.30	99.90	99.43
0.2	99.20	99.10	98.80	99.20	99.08
0.5	97.50	97.80	97.30	97.70	97.58

**Table 12:** Impact of soft label weighting factor (α) on average accuracy (%) and standard deviation across PU dataset transfer tasks.

α Value	Avg. Accuracy (%)	Std. Dev.
0.3	98.75	0.42
0.5 (default)	99.43	0.28
0.7	99.21	0.31
0.9	98.92	0.38

## Data Availability

The dataset used in this article can be obtained from the corresponding author upon request.
